# Neural mechanism of dopamine modulating singing related behavior in songbirds: an updated review

**DOI:** 10.7717/peerj.19500

**Published:** 2025-06-05

**Authors:** Linghua Zhong, Weiting Li, Mengjiao Liu, Wei Sun, Zhibin Liu, Songhua Wang

**Affiliations:** 1 School of Sports and Health, Jiangxi Science and Technology Normal University, Nanchang, China; 2 Jiangxi Provincial Key Laboratory of Organic Functional Molecules, Jiangxi Science and Technology Normal University, Nanchang, China

**Keywords:** Dopamine, Songbird, Singing behavior, Song control system

## Abstract

Similar to human language, songbird singing is a complex motor skill learning behavior that is regulated by an interconnected network of neural nuclei in the brain. This network of nuclei demonstrates structural homology with human vocal control-related brain regions and shares common regulatory mechanisms for vocal learning. As an important neurotransmitter, dopamine plays a key role in the learning and maintenance of songbirds’ singing behavior. Studies have demonstrated that the dopaminergic system plays a critical role in regulating the plasticity of singing via the midbrain dopamine pathway, which projects to the song control circuit. Novel experimental techniques, such as optogenetic circuit manipulation and neural activity monitoring, have significantly advanced our understanding of the cellular and synaptic mechanisms underlying vocalization behavior of dopamine effects. This review offers an updated insight into the neural mechanisms by which dopamine modulates singing-related behavior, along with future prospects for utilizing dopamine in the treatment of speech-related disorders.

## Introduction

It is a well-established tradition in the field of neurophysiology to utilize model organisms for scientific investigation. From nematodes and fruit flies, to mice and monkeys, these models have made significant contributions towards elucidating neural mechanisms underlying behavior. Among avian species, songbirds such as the zebra finch and canary are widely acknowledged as invaluable model organisms that have facilitated important advancements in neurophysiology, developmental biology, language processing, and beyond (Wang, Li & Meng, 2022). Apart from humans, songbirds are among the few vertebrates that possess intricate vocal learning abilities. Due to the inherent limitations in studying the human brain, they serve as a captivating neurological model for investigating the mechanisms underlying language acquisition and production through comparative physiology (Doupe & Kuhl, 1999). Both of songbird and human need auditory feedback to participate in the process of vocal learning (Prather, Okanoya & Bolhuis, 2017), and have stronger vocal learning ability in the juvenile stage and control ability of complex acoustic structures and syllable sequences (Doupe & Kuhl, 1999). Songbirds offer a powerful comparative model system for exploring how the nervous system integrates motor and sensory information to facilitate learning and control (Murphy et al., 2017). Song can be easily quantified and manipulated to facilitate experimentation and assessment of the relation between behavior and the associated neural activity (Gadagkar & Goldberg, 2013; Prather, 2013; Gadagkar et al., 2016; Duffy et al., 2022; Zemel et al., 2023).

The song learning process of songbirds consists of two periods: the sensory phase and the sensorimotor phase. During the sensory stage, juvenile birds create a memory template after being exposed to their tutor’s song. In the sensorimotor period, juvenile birds rely on auditory feedback to match their songs with the memorized template and continuously refine their vocalizations until they reach a relatively stable state known as crystallization (Brainard & Doupe, 2002; Zhang et al., 2023). According to research needs, the mechanism of singing behavior in songbirds is primarily investigated using zebra finches (Zhang et al., 2023) and bengalese finches (Zhao et al., 2018), canaries (Cohen et al., 2020) and European starlings (Riters et al., 2014b). Recently, oscine-net.org has provided unprecedented access to detailed songbird connectivity data, fostering insights into the neural circuits that underlie complex behaviors (Savoy, Anderson & Gogola, 2024).

In recent decades, there has been a surge of research investigating the neural mechanisms underlying dopamine (DA) regulation of singing and social behaviors (Chen & Goldberg, 2020; Wood, 2021; Das & Goldberg, 2022). The development of new tools for circuit manipulation and monitoring neural activity has greatly enhanced our understanding of these cellular and synaptic mechanisms. DArgic inputs to the striatum play a role in learning of fine motor skills involved in vocal performance (Hoffmann et al., 2016) song learning (Xiao et al., 2018) and encode performance errors (Gadagkar et al., 2016) in songbirds. DA is a key substance in the brain that regulates learning and motivation (Wise, 2004; Mohebi et al., 2019). With the deepening of research, the understanding of the role of DA is constantly expanding (Gershman et al., 2024). Similar to other animal motor learning and human language learning processes, the song learning behavior of songbirds also requires the involvement of DA system regulation. Therefore, the song learning behavior of songbirds can provide an excellent research model for exploring the mechanisms of DA system regulation of complex motor control and learning, including human language (Chen et al., 2019; Chen & Goldberg, 2020; Barr, Wall & Woolley, 2021; Wood, 2021). Birdsong neurobiologists provided valuable clues for revealing the neural mechanisms by which the DA system regulates the singing control system and singing behavior of songbirds (Duffy et al., 2022; Ben-Tov, Duarte & Mooney, 2023; Ramarao et al., 2023; Roeser et al., 2023). However, there are still open questions about how DA acts in Area X and there are some but fewer studies about DA in the high vocal center (HVC) and robust nucleus of the arcopallium (RA).

Here, we focus on the neural mechanisms underlying DA’s modulation of singing related behavior in songbirds. Additionally, we will discuss how research on DA in songbirds may inform our understanding of human speech in future work.

## Survey Methodology

This review summarizes the neural mechanisms underlying DA modulation of singing-related behaviors, including song learning, production, and sexual motivation in songbirds. Articles were sourced from the Web of Science, Scopus, and PubMed databases using the following search terms: (Dopamine AND Songbird). A preliminary search conducted between 1994 and 2024 identified 187 articles. And added search terms: (Dopamine AND human AND songbird), a preliminary search conducted between 1994 and 2024 identified 32 articles. After removing duplicates and irrelevant literature on singing behavior or human speech, and conducting a thorough screening process through abstracts and full-text reviews, a total of 124 articles were selected for analysis.

## A brief overview of song system anatomy

**Figure 1:**
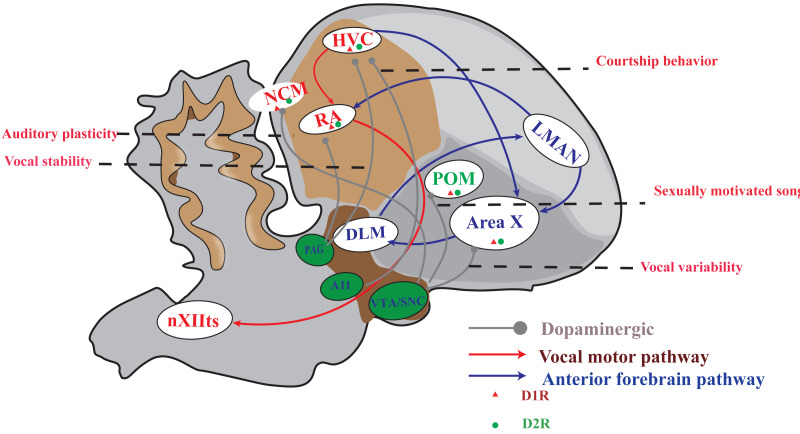
A simplified diagram of the song control system, DA receptor distribution and dopaminergic projection in songbirds. The songbird’s singing control system comprises two distinct pathways: the vocal motor pathway (VMP) and the anterior forebrain pathway (AFP). The VMP encompasses a sequential connection of HVC → RA → nXIIts, while the AFP involves HVC → Area X → DLM → LMAN. Notably, dopamine receptors are prominently distributed in Area X and moderately distributed in HVC, RA, LMAN, DLM, and POM. Furthermore, projections from the VTA/SNc complex primarily target Area X and POM; however, both HVC and RA receive projections from both the VTA/SNc complex as well as PAG. A11 projected to HVC.

The songbird brain possesses a specialized network of structures, known as the song system, which is dedicated to imitative vocal learning and production. This system comprises two distinct neural pathways (Fig. 1). One of the pathways is the vocal motor pathway (VMP), which shares similarities with the human motor cortex-brainstem pathway (Jarvis, 2007; Cahill et al., 2021; Zhang et al., 2023). This particular pathway originates from HVC, a premotor nucleus that are similar to those of human laryngeal motor cortices layers 2 or 3 (Pfenning et al., 2014; Jarvis, 2019), to the RA, which is similar to the human laryngeal motor cortices layer 5 neurons (Jarvis, 2004; Jarvis, 2019), and then to the tracheosyringeal part of the hypoglossal nucleus (nXIIts), which dominate the vocal muscle, and ultimately producing singing behavior. The other is the anterior forebrain pathway (AFP), which is similar to the cortical-basal ganglia-thalamus-cortical circuit in human. This pathway is projected from HVC to Area X, then to the medial portion of the dorsolateral nucleus of the anterior thalamus (DLM), and finally to the lateral magnocellular nucleus of the anterior nidopallium (LMAN). AFP signal is output to RA through LMAN, and VMP mainly controls the output of singing. AFP is related to the song learning of young songbirds and the song plasticity of adult songbirds (Brainard, 2004; Doupe et al., 2004; Xiao & Roberts, 2021).

## DArgic system and DA innervation in mammalians and songbirds

### The effects of DA and dopaminergic distribution

DA, the main catecholamine neurotransmitter in the brain, is predominately produced in the midbrain and released to various brain regions including the cortex, dorsal striatum and nucleus accumbens (Costa & Schoenbaum, 2022). DA’s effects are widespread and include modulation of a number of reward, motor, cognitive, motivational, addiction, learning, olfaction, vision, hormonal regulation, sympathetic regulation and other activities (Gale, Person & Perkel, 2008; Beaulieu, Espinoza & Gainetdinov, 2015; Turk et al., 2021; Costa & Schoenbaum, 2022). DA can modulate behavior of rodents through regulating the fast neurotransmitters glutamate and gamma-amino butyric acid (GABA) (McCutcheon, Krystal & Howes, 2020; Ghanavati et al., 2022; Li, Wang & Gao, 2012). DA binding to DA receptors can alter the phosphorylation of critical subunits of glutamate AMPA receptors (GluA1) and NMDA receptors (GluN2B) (Dell’anno, Pallottino & Fisone, 2013; Hobson et al., 2013; Song et al., 2013; Flores-Barrera et al., 2014; Murphy et al., 2014). Recent reserch showed that glutamate release from ventral tegmental area (VTA) glutamate neurons play positively reinforcing role, but that DA release from VTA glutamate neurons participate in avoidance behavior in mice (Warlow et al., 2024).

Most of the DA in the mammalian brain is produced by a subset of neurons located in the ventral midbrain, namely the VTA and the substantia nigra pars compacta (SNc) (Costa & Schoenbaum, 2022). DArgic midbrain target the cortex, nucleus accumbens, and dorsal striatum, forming the mesocortical, mesolimbic, and nigrostriatal pathways, respectively in mammalians (Costa & Schoenbaum, 2022). DArgic neurons in the midbrain of songbirds are mainly distributed in the VTA/SNc complex, the periaqueductal gray (PAG), midbrain central gray (CG) and A8-A15 of songbirds (Soha, Shimizu & Doupe, 1996; Appeltants et al., 2000; Gale & Perkel, 2005; Goodson et al., 2009; Kelly & Goodson, 2015). These DArgic neurons project to the singing control nucleus including Area X, RA and HVC, which affects the learning and production of singing. The striatal song nucleus Area X in songbirds receives even more dense DArgic innervation from VTA and SNc relative to the surrounding striatum (Lewis et al., 1981; Soha, Shimizu & Doupe, 1996; Cragg, 2005; Gale & Perkel, 2005). HVC primarily receives DArgic projections from PAG, with a minor contribution from VTA (Appeltants et al., 2000). The surrounding telencephalon appears to receive projections from SNc and its rostral extension (Appeltants et al., 2000). RA receives DArgic inputs from the PAG and VTA DArgic cell groups (Appeltants et al., 2000; Appeltants, Ball & Balthazart, 2002).

### Dopamine receptors types and distribution

DA receptors are widely expressed in the central nervous system, and there are currently five known subtypes: D1, D2, D3, D4, and D5. These five DA receptor subtypes are divided into two categories based on their structural and pharmacological characteristics, and all belong to the superfamily of G protein-coupled receptors (GPCRs), including D1-like receptors and D2-like receptors. Among them, D1-like receptors include D1 and D5, while D2-like receptors include D2, D3, and D4 (Kebabian, 1978; Spano, Govoni & Trabucchi, 1978; Beaulieu, Espinoza & Gainetdinov, 2015).

In the mammalian central nervous system, D1 and D5 receptors are generally located in postsynaptic DA mediated cells, D2 and D3 have presynaptic and postsynaptic cellular localization (Beaulieu & Gainetdinov, 2011; Baik, 2013), and D4 receptors are highly expressed in the retina (Cohen et al., 1992). Among them, D1-like receptors(D1Rs) are mainly located in the caudate putamen (striatum), nucleus accumbens, substantia nigra, olfactory bulb, amygdala and frontal cortex (Wamsley et al., 1989), and D2-like receptors (D2Rs) are mainly expressed in the striatum, lateral part of the globus pallidus, nucleus accumbens, ventral tegmental area, hypothalamus, amygdala, cortex, hippocampus and pituitary (Yokoyama et al., 1994). Different subtypes of receptors have different physiological functions. D1, D2 and D3 receptors play a critical role in the regulation of motor function. Presynaptic self receptors, mainly D2 receptors, are also involved in the regulation mechanism of neuronal excitability, DA synthesis and phased release in response to changes in the extracellular level of neurotransmitters (Missale et al., 1998; Beaulieu & Gainetdinov, 2011). D1 and D2 receptors can regulate the role of DA in learning and memory, and studies have shown that DA can regulate the activity of prefrontal cortical neurons and promote the content of visuospatial working memory in memory through D1Rs (Sawaguchi, 2001).

Within the zebra finch, there exist two types of DA receptors: D1Rs, which include D1A, D1B and D1D; and D2Rs, which include D2, D3 and D4 (Kubikova, Wada & Jarvis, 2010). The striatum exhibits high expression levels of both the D1A and the D1B receptor subtypes as well as the D2Rs subtype (Kubikova, Wada & Jarvis, 2010). The expression of D1Rs in song nuclei was significantly higher than that in the surrounding regions. Moreover, the developmental regulation of D1R expression showed a decrease during both sensory acquisition and sensorimotor phases of song learning. In Area X, half of the cells expressed both D1ARs and D2Rs (Kubikova, Wada & Jarvis, 2010). In HVC, the majority of cells expressing the D1Rs and D2Rs subtypes were vesicular glutamate transporter 2(VGLUT2)-positive, whereas colocalization with Vglut2 occurred in few cells in Area X and in 70 percent of cells in PAG (Haakenson et al., 2024). DARs are also highly expressed in the medial preoptic nucleus (POM) (Hahn et al., 2019). Studies conducted by Kelm-Nelson et al. (2013) and Riters et al. (2014a) have demonstrated the involvement of POM in the motivation to sing among European starlings. As a crucial brain site, the POM nucleus activates the appetitive aspects of sexual behavior in songbirds. Tyrosine hydroxylase (TH), which is the rate-limiting enzyme in catecholamine synthesis (including DA) in the brain, was found to be abundant in Area X in house crows. While staining in RA and HVC was less prominent, these findings suggest that DA plays a crucial role in Area X of songbirds (Sen et al., 2019).

DARPP-32, a phosphoprotein regulated by both DA and cAMP, is primarily found in DArgic neurons and is associated with postsynaptic targets of DA. In vertebrates, the expression of DARPP-32 is highest in both the medial and lateral striatum. Further investigation revealed that axon terminals rich in DARPP-32 were present in DArgic neurons of the VTA/SNc complex, which subsequently projected to the striatum in songbirds (Singh & Iyengar, 2019). Overall, the expression of DARPP-32 appears to be increased in regions involved in sensory information integration, providing further evidence for its role as a molecular integrator of diverse signal processing pathways. Recent research showed that DARPP-32-labeled DArgic neurons of the caudolateral nidopallium participated in visual discrimination in corvids (the family Corvidae is known for its cognitive abilities, akin to those of great apes) (Parishar, Rajagopalan & Iyengar, 2024).

## DA Modulating Singing Related Behavior

### DA-induced plasticity in the auditory cortex of songbird

In songbirds, the majority of auditory areas studied thus far are situated in the caudomedial forebrain and encompass the thalamo-recipient field L (subfields L1, L2, and L3), as well as the caudomedial and caudolateral mesopallium (CMM and CLM, respectively) and the caudomedial nidopallium (NCM) (Pinaud & Terleph, 2008). D1Rs are abundant in both NCM and CMM, while D2Rs are only found in high levels in CMM but not in NCM. The expression of both D1Rs and D2Rs is absent in Field L (Cragg, 2005). In female white-throated sparrows, exposure to conspecific song resulted in increased activation of TH fibers and elevated levels of DA metabolites in the NCM (Matragrano et al., 2012). Activation of D1Rs induces plasticity in the auditory pallium of songbirds by reducing the amplitude of sIPSCs and glutamatergic sEPSCs, while increasing the frequency of sEPSCs in NCM. In an *in vivo* experiment, activation of D1R reduces firing activity in putative interneurons but increases it in putative excitatory neurons of NCM (Macedo-Lima, Boyd & Remage-Healey, 2021).

Barr, Wall & Woolley (2021) demonstrated that the combination of passive song playback and pharmacological manipulation of DA in the NCM induces enduring changes in female zebra finches’ song preferences, suggesting a direct role for DA in modulating the incentive salience of communication signals within sensory processing areas. The results of another study demonstrated that exposure to courtship songs, but not non-courtship songs, resulted in an upregulation of c-FOS expression, an activity-dependent immediate early gene, in DA neurons located in the VTA (Nordeen, Holtzman & Nordeen, 2009). This increase was found to be contingent upon whether female subjects had been exposed to adult songs during their developmental stages. The aforementioned findings highlight the long-lasting impact that early auditory experiences can have on both the quantity and sensory reactivity of populations of DA neurons (Barr & Woolley, 2018). Furthermore, developmental exposure to music also lead to structural brain changes in motor and auditory areas in early childhood (Hyde et al., 2009).

### Effect of DArgic system on song learning

In the past few decades, DA has been extensively implicated in learning. The idea that DA provides a learning signal fits beautifully with the literature that DA modulates synaptic plasticity in the striatum, the primary forebrain target of DA (Berke, 2018; Hisey, Kearney & Mooney, 2018; Xiao et al., 2018; Daou & Margoliash, 2020). Hoffmann et al. (2016) demonstrated that lesions of the DAergic inputs to Area X in adult male Bengalese finches significantly reduced the magnitude of vocal learning driven by disruptive auditory feedback in a negative reinforcement task. Furthermore, lesions of DArgic terminals within Area X in adult male Bengalese finches had a twofold effect on song learning behavior. Firstly, over the course of several days, lesioned birds exhibited a systematic decrease in the fundamental frequency (pitch) of syllable regardless of auditory feedback. Secondly, these same lesions resulted in significant deficits in sensorimotor learning when exposed to pitch-shifted feedback (Saravanan et al., 2019). In juvenile zebra finches, depletion of DA in Area X during the critical period for song learning results in impaired imitation of the tutor’s song (Hisey, Kearney & Mooney, 2018). In addition, VTA_X_ neurons (known to be DArgic) are essential for internally guided vocal copying in juvenile songbirds and externally reinforced forms of vocal learning in adults male zebra finches (Hisey, Kearney & Mooney, 2018). Moreover, D1Rs signaling in Area X is a critical component of both juvenile song imitation and adult pitch learning in zebra finches (Hisey, Kearney & Mooney, 2018).

In adult male zebra finches, All VTAerror neurons were phasically suppressed by distorted auditory feedback (DAF) during singing (Gadagkar et al., 2016). In juvenile zebra finches, song tutoring increased the level of immediate early gene in VTA DArgic neurons (Chen, Matheson & Sakata, 2016). Yanagihara et al. (2021) found that a subset of VTA/SNc units exhibited phasic activity precisely time-locked to the onset of the song bout, specifically at the beginning of songs in juvenile male zebra finches. The results indicate that the phasic activity in the VTA/SNc serves as an initiating signal for song vocalization (Yanagihara et al., 2021). Kearney et al. (2019) utilized song performance-contingent optogenetic stimulation of VTA afferents from AIV/VP, which were sufficient to drive pitch learning of zebra finches. In the sensorimotor period, the DArgic neurons in songbirds encode signals for evaluating songs that resemble reward prediction errors (RPEs) (Chen & Goldberg, 2020). In the songbird, the RPE represents the discrepancy between the perceived and predicted quality of a syllable based on recent practice (Chen & Goldberg, 2020). Area X-projecting DArgic neurons in songbirds exhibit movement-related activity exclusively outside of singing, and RPE-like activity solely during singing (Chen et al., 2021).

The acquisition of skills requires the assessment of motor output against internal performance benchmarks. A pathway originating from a high-order auditory cortical region, the ventral intermediate arcopallium (AIV), projects to the ventral tegmental area and is essential for song learning (Mandelblat-Cerf et al., 2014; Chen et al., 2019; Kearney et al., 2019). This pathway sends error signals to VTA (Mandelblat-Cerf et al., 2014). Ventral pallidum (VP) neurons encode both song performance errors and precise song timing, thereby transmitting error and error prediction signals to the VTA (Chen et al., 2019). Furthermore, the modulation of VTA_X_ firing by both AIV and VP lends support to the hypothesis that signals associated with song evaluation can reach Area X *via* the VTA (Gale & Perkel, 2010). In songbirds, the subthalamic nucleus (STN) projects to VTA, and micro-stimulation of STN can effectively excite VTA neurons. Moreover, a small subset of STN neurons exhibit precise timing in song production and performance error signals, indicating that the pathway from STN to VTA plays a crucial role in song learning (Das & Goldberg, 2022).

Inhibiting DA signaling in the HVC of juvenile zebra finches during tutoring results in the failure to imitate tutor songs, while stimulating PAG terminals in the HVC and playing tutor songs through a speaker is sufficient to induce young zebra finches to copy their tutors (Tanaka et al., 2018). The facilitated learning is assumed to be due to increased attention (Tanaka et al., 2018). Attention-associated motor skill learning is regulated by DA (Liu et al., 2021). The attentive and responsive approach of juvenile songbirds towards their tutor is facilitated by the binding of DA to D1 receptors (Liu et al., 2021).

### Effect of DArgic system on singing production

Songbirds produce two types of songs: directed song (FD), which is aimed at attracting female mates, and undirected song (UD), which is not oriented towards females. Depletion of DArgic nerve terminals in Area X results in an decreases vocal variability during UD but not FD song (Miller et al., 2015). When male birds sing in the presence of a female, a social context associated with decreased basal ganglion (BG)-induced song variability, the extracellular DA level is increased in the avian BG nucleus Area X (Leblois, Wendel & Perkel, 2010). These results suggest that DA could trigger song variability changes through its action in Area X (Leblois, Wendel & Perkel, 2010).

When a D1R antagonist is administered into Area X, it alters neuronal firing patterns and induces greater pitch (fundamental frequency) variation in FD songs, resembling that of UD songs (Sasaki et al., 2006; Liu et al., 2021). The mean accuracy of the song motif, which one of the parameters of song stability, exhibited a negative correlation with D1AR expression levels, whereas the sequential match of song motif displayed a positive correlation with D2R expression levels in Area X as compared to the surrounding striatum (Bosíková et al., 2012). Disruption of D1R transmission in Area X abolished social context-related alterations in song variability (Leblois, Wendel & Perkel, 2010). Activation of D1R in the basal ganglion decreases syllable variability, while it does not participate in regulating song timing (Leblois & Perkel, 2012). The intrinsic motivation underlying undirected song in zebra finches is critically regulated by DA through D2Rs, with activation of these receptors leading to a dose-dependent decrease in the latency of the first song (Leblois & Perkel, 2012). D3 receptor agonist can reduce cell death caused by Area X injury in zebra finch and lead to longer song motif (Lukacova et al., 2016).

Other studies have shown that the VTA/SNc played distinct roles in song maintenance depending on social context, with unilateral lesions of the VTA/SNc resulting in a decrease stability of song motif of FD among male zebra finches but not in UD (Hara et al., 2007). Additionally, the firing rate of VTA neurons exhibited contextual differences in social settings (Yanagihara & Hessler, 2006). Manipulation of VTA axon terminals in Area X *via* optogenetic excitation and inhibition induced learned alterations in pitch, resulting in subsequent performances of targeted syllables with enhanced proficiency (Xiao et al., 2021). In song learning, Area-X-projecting VTA (VTA_X_) neurons signal errors in predicted song quality. To represent performance prediction errors, songbird DA neurons must compute the difference between the actual “just heard” error and the error that was predicted at that specific time step of the song. The ventral pallidum sends both error and error prediction signals to the VTA in songbirds (Chen et al., 2019). So, the ventral pallidum sending signal to VTA played key role in birdsong learing. In addition, Duffy et al. (2022) reports the highest levels of DA spikes occur at specific vocal targets, indicating either active maintenance of an existing song or a shift towards a nearby variant. These findings demonstrate that spontaneous DA spikes can accurately reflect natural fluctuations in behavior, independent of experimental cues or rewards (Duffy et al., 2022).

Phasic increases in DA levels during singing lead to the repetition of song syllables, thereby also hindering the smooth initiation and termination of birdsong (Xiao et al., 2021). The recent research found that midbrain cell group (A11) neurons, which is part of a larger DArgic neuronal network paticipated FD calls of zebra finches and A11 projecting to HVC playing key role in courtship behaviors related FD singing (Ben-Tov, Duarte & Mooney, 2023).

### DA effects on the sexual motivation in songbirds

The DAergic system plays a crucial role in regulating female songbird’s sexually-motivated responses to male courtship songs. Peripheral administration of a non-selective DA reuptake inhibitor resulted in increased responsiveness of female starlings to non-biologically relevant male purple martin songs (Ben-Tov, Duarte & Mooney, 2023). Another study demonstrated paired (in cage with male and female) zebra finches exhibited elevated levels of DA and its metabolite in the ventral medial striatum, where the nucleus accumbens is located, compared to unpaired birds (paried male VS unparied male; paried female VS unparied female) (Banerjee et al., 2013). Moreover, a higher percentage of DArgic neurons expressing the immediate early gene *Fos* was observed in the VTA of paired zebra finches than unpaired ones, suggesting a potential involvement of the mesolimbic DArgic pathway in pair formation and indicating a conserved neural mechanism underlying monogamy across avian and mammalian species (Banerjee et al., 2013). In addition, percentages of TH^+^ Fos-expressing neurons were higher in the ventral medial striatum, caudal A11 nucleus and rostral A11 nucleus of subjects in the paried male group than in subjects of the unpaired male group (Banerjee et al., 2013). Day et al. (2019) discovered that the activation of DA through the D2 receptor, but not the D1 receptor, can elicit a song preference in unpaired female finches. Additionally, blocking the D2 receptor eliminated song preference in paired females.

DA in the POM plays a complex modulatory role in the production of sexually-motivated song,and optimal levels of D1 receptor stimulation are necessary to facilitate singing behavior of male European starlings (Riters et al., 2014a). Activation of D1 receptors in the POM in male European starlings triggers sexually motivated and conditioned place preferences induced by hearing female European starlings’ songs (DeVries et al., 2015). In addition, stimulating D1 receptors in the POM is most effective in facilitating sexually-motivated singing behavior (DeVries et al., 2015). These findings provide evidence for a context-specific central role of the POM in vocal communication and highlight the intricate modulatory effect of D1 receptors on sexually motivated behavior (Maksimoski et al., 2023).

Tokarev et al. (2017) discovered that the DArgic reward circuitry in zebra finches can promote both social cohesion and breeding boundaries simultaneously. Interestingly, unmated male birds showed increased striatal DA neurotransmission after hearing courtship songs, unfamiliar song playbacks were also be strongly reinforced. However, unmated females did not exhibit these responses. The song reinforcment in unmated male were dependent on striatal D2 receptors. In mated females, the mate song was found to be a strongly reinforcing stimulus with high specificity. However, only marginally higher levels of dopamine neurotransmission were observed in response to the mate song compared to non-mate songs. These findings suggest that song-induced activation of the DArgic system serves a dual purpose in social songbirds: as a low-threshold social reinforcement for males and an ultra-selective sexual reinforcement for females (Tokarev et al., 2017). When engaging in courtship behavior with a female, the motivation to seek water was reduced and the dopamine responses to both water availability and performance outcomes of song were diminished. Instead, DA signals in Area X were primarily influenced by female vocalizations synchronized with the courtship song. The dopamine system effectively managed concurrent motivations by directing vocal performance and social feedback signals to a striatal area for communication purposes, while also flexibly adjusting reward expectations based on the prioritized drive (Roeser et al., 2023).

Individuals with greater competitive ability tend to dominate their opponents and secure priority access to resources, including food, territory, and mates. In mate competition, male zebra finches with high competitive ability (HCA) exhibit increased levels of tyrosine hydroxylase mRNA in the VTA and D1R mRNA in the POM compared to low competitive ability (LCA) males. Additionally, HCA males demonstrate decreased D1R mRNA expression in the anterior hypothalamus relative to LCA males. These findings suggest that region-specific alterations in DA gene expression areas related to competitive ability during mate competition (Eswine, Pontinen & Heimovics, 2019).

### Effect of DA on electrophysiological activity of singing control nucleus

In Area X, the excitability of spiny neurons which with prominent dendritic spines, is modulated by DA, with D1R enhancing excitability and D2R reducing it (Ding & Perkel, 2002). Furthermore, activation of D1Rs leads to a decrease in excitatory synaptic transmission in Area X spiny neurons. This effect occurs at a presynaptic site and can be mimicked by adenylyl cyclase activation but blocked by protein kinase A (PKA) inhibitors and adenosine A1 receptor antagonists (Ding & Perkel, 2002) DA can precisely regulate information processing through spiny neurons in Area X, thereby modulating song learning and maintenance (Ding, Perkel & Farries, 2003). Our previous study found that DA significantly enhanced the excitability of RA projection neurons. Moreover, D1R agonists mimic the effects of DA on RA projection neuron excitability, while D2R agonists has no impact. These findings suggest that the binding of dopamine to D1 receptors increases the excitability of RA projection neurons (Liao et al., 2013). The further studies find out that activation of D1 receptors can enhance NMDA-induced gain modulation through a PKA-dependent pathway (Wang et al., 2015). Furthermore, DA reduces the frequency of spontaneous and miniature excitatory postsynaptic currents (sEPSCs and mEPSCs) in the RA (Wang et al., 2020). The effects of DA on excitatory synaptic transmission in the RA of adult male zebra finches are mediated by D1 receptors, as demonstrated by the ability of a D1 receptor agonist to mimic these effects while a D2 receptor agonist does not (Wang et al., 2020).

D1R activation leads to a decrease in the amplitudes of sEPSC and spontaneous inhibitory postsynaptic currents (sIPSC), while increasing the frequency of sEPSC in NCM (Macedo-Lima, Boyd & Remage-Healey, 2021). Additionally, D1R activation reduces the firing rate of putative inhibitory neurons but increases that of putative excitatory neurons . Moreover, D1R activation results in flattened stimulus-specific adaptation slopes of NCM neurons (Macedo-Lima, Boyd & Remage-Healey, 2021). These results indicated that D1R inhibited the synaptic transimission in NCM.

DA in the basal ganglia decreased trial-to-trial neural variability when birds engage in courtship song. In this study, Budzillo et al. (2017) provide evidence for DA binding to DA receptor modulates the coupling of excitatory and inhibitory events *in vitro*, leading to a dynamic alteration in the synchrony of a modeled population of basal ganglia output neurons receiving both types of inputs. Therefore, the excitatory interneuron serves as a biophysical mechanism to introduce or modulate neural variability within this circuit, playing a vital role in its function (Budzillo et al., 2017).

## Human Speech, Songbird Vocalizations and Dopamine

Some study showed that speech-induced phasic DA release into the dorsal striatum and speech motor cortex exerts direct modulation of neuronal activity in these regions and drives left-hemispheric lateralization of speech production network in human (Fuertinger et al., 2018). The neural mechanism of DA regulation of human speech remains to be further studied. Although there are differences in the nervous system of vocal control between humans and songbirds, given the limitations in studying human language and the ethical constraints involved, we primarily investigate the mechanism by which DA regulates human speech using songbirds as a model (Simonyan, Horwitz & Jarvis, 2012). The singing of songbirds are similar to the vocalization of human speech. They both have complex vocalization structures, and they are all vocalized by learnering from adult “tutors” in their infancy (Brainard & Doupe, 2013), and is stably maintained through sensory motor error correction throughout life.

In songbirds, DA system is closely related to reward learning (Chen & Goldberg, 2020). As the main communication mode of birds, singing is associated with the reward mechanism of DA, especially in courtship, territorial defense or social interaction. When a singing bird unexpectedly hits the right note, its DA neurons are activated, and DA signals reinforce vocal variations (Chen & Goldberg, 2020). The acquisition of human speech is also a result of reinforcement learning (Toutounji et al., 2024). Further research is needed to determine whether DA plays a role in human speech .

Single-cell RNA sequencing technology has become the state-of-the-art approach for unravelling the heterogeneity and complexity of RNA transcripts within individual cells, as well as revealing the composition of different cell types and functions within highly organized tissues/organs/organisms (Jovic et al., 2022). The inter-species differences in germ cell development between chicken and zebra finch wereinvestigated by single-cell RNA sequencing (Jung, Seo & Han, 2023; Biegler et al., 2025). Colquitt et al. (2021) used single-cell RNA sequencing technology to detect the HVC and RA nuclei of Bengal finch and zebra finch and compared them with neocortex of mice. They found that the neural transcription characteristics of the vocal circuits in songbirds are similar to those in the mammalian neocortex, but their developmental origins differ. In the future work, Single-cell RNA sequencing technology can be used to o characterize the neuronal repertoire of SNc /VTA in DA neurotransmitter rich regions and DA regulated brain Area-X region, so as to further compare the regulatory effects of dopamine in songbird singing and human language behavior.

## Conclusion

DA paticipated in auditory perception, song learning and production, sexual motovation, female-directed calls and singing related courship behaviors. With the deepening of research, the understanding of the role of DA is constantly expanding (Gershman et al., 2024). Continued research progress in songbird is crucial for advancing understanding of how the DA system evolved to shape the diverse array of brain structures and behaviors among the vertebrate lineage (Macedo-Lima & Remage-Healey, 2021). The development and function of midbrain DA neuron subtypes in songbirds need further studied (Garritsen et al., 2023). Whether there are new nuclei or circuits involved in the regulation of DA on singing behavior. These findings not only deepen the understanding of the neural mechanism of vocal learning in birds, but also provide an evolutionary perspective for the study of human speech production and disorders.

## Additional Information and Declarations

### Data Availability

The following information was supplied regarding data availability: This is a literature review.
